# Restoration of Muscle Function Following Distal Biceps Tendon Reinsertion: A Narrative Review

**DOI:** 10.3390/jcm15062430

**Published:** 2026-03-22

**Authors:** Michał Harasymczuk, Ewa Bręborowicz, Aleksandra Bartkowiak-Graczyk, Anna Madziewicz, Tomasz Balcerek, Leszek Romanowski

**Affiliations:** 1 Traumatology, Orthopedics and Hand Surgery Department, Dega Orthopedics Institute, Poznań University of Medical Sciences, PL-61-545 Poznan, Poland; ewabreborowicz@ump.edu.pl (E.B.); ale.bartkowiak@gmail.com (A.B.-G.); annamadziewicz1@gmail.com (A.M.); lromanowski@orsk.pl (L.R.); 2 Department of Physiotherapy, Poznań University of Medical Sciences, PL-61-545 Poznan, Poland; 3 Department of Gynecology Obstetrics and Gynecologic Oncology, Gynecological Obstetric Clinical Hospital of Poznań University of Medical Sciences, PL-60-535 Poznan, Poland; tomaszbalcerek@gmail.com

**Keywords:** distal biceps tendon rupture, muscle function restoration, supination and flexion strength, surgical approaches, comparative analysis, functional outcomes, evidence-based practice

## Abstract

**Background/Objectives**: Distal biceps tendon rupture (DBTR) significantly impairs upper-limb function, particularly in movements requiring elbow flexion and forearm supination. This condition continues to attract clinical interest due to its complex biomechanics, evolving surgical strategies, and the growing emphasis on comprehensive rehabilitation. Contemporary evidence highlights the value of a multidisciplinary approach that integrates precise surgical repair with structured, progressive physiotherapy to optimize outcomes effectively. **Methods**: We performed a comprehensive review of the literature by searching PubMed/MEDLINE, and a narrative review format was adopted to synthesize the available evidence. **Results**: Studies comparing single-incision and double-incision techniques show that both achieve excellent outcomes, although the decision should be tailored to patient-specific factors, surgeon expertise, and the reported complication risk, which may vary between 5% and 63%. Regardless of technique, restoring tendon integrity is essential for regaining normal strength and supination capability. Rehabilitation following DBTR repair relies on a phased and carefully monitored program. Early physiotherapy focuses on a controlled range of motion and the prevention of stiffness while protecting the repair. As healing progresses, strengthening exercises targeting the biceps, triceps, and brachialis are introduced, alongside endurance training to enhance overall functional capacity. Evidence strongly supports early mobilization protocols, where active motion and graded resistance are initiated within the first postoperative week, resulting in faster and more complete functional recovery compared to prolonged immobilization. **Conclusions**: Long-term outcomes after DBTR repair are consistently favorable. Most patients return to full activity or sport at an average of 5.4 months, although timelines vary with rehabilitation intensity and baseline fitness. Notably, 93–100% recover their pre-injury activity level, including participation in competitive sports.

## 1. Introduction

The distal biceps tendon is essential in maintaining the functional integrity of the elbow joint, facilitating both flexion and supination of the forearm. Ruptures (avulsions) of the M. biceps brachii distal attachment are rare injuries occurring in 2.55 to 5.35 per 100,000 patients/year [1], accounting for 3 to 12% of all M. biceps brachii tendon injuries [2,3]. In most orthopedic literature, distal biceps tendon ruptures (DBTRs) occur in 56–86% of cases in the dominant arm of middle-aged men [4,5,6,7], mainly in their fourth and fifth decades of life [8]. A limited number of documented DBTR cases have been noted within the female population [9]. A larger cross-sectional area (CSA) of the M. biceps brachii and a deficit of female hormones in men may contribute to the sex differences in DBTR occurrence [10]. A recent systematic review reported a 96.7% incidence of DBTR among males, with a mean age of 46.8 years. Furthermore, it was noted that 94.7% of these individuals engaged in an active lifestyle (either as professionals or as a leisure pursuit). In comparison, 68.2% were employed in either a physical or hybrid physical occupation [11].

The reports concerning the surgical treatment of DBTR have indeed surfaced in the medical literature with remarkable frequency since the 1970s, indicating a growing awareness and interest in this particular injury [12]. The initial documentation of the first cohort of patients affected by this specific pathology, in whom surgical intervention was not carried out, sparked an unexpected wave of attention and extensive discussions within the medical community [13]. On one side of the debate, it was suggested that this trend was linked to the long-standing neglect of such an injury and the limited array of current diagnostic techniques available for a thorough evaluation of the condition. Conversely, this situation advocated for further comparative studies focused on assessing the effectiveness of rehabilitation methods versus examining how the absence of surgical intervention can impact arm function in affected patients [14,15].

The restoration of muscle function, including peak strength, range of motion (ROM), and endurance following DBTR, is a critical area of study within sports medicine and rehabilitation, as it directly impacts the functional recovery of athletes and active individuals. Understanding the mechanisms behind effective rehabilitation protocols can lead to improved outcomes and a quicker return to sport, thereby enhancing overall athletic performance and reducing the risk of re-injury. Research has shown that a combination of strength training, flexibility exercises, and sport-specific drills can significantly improve recovery, allowing individuals to regain their pre-injury biomechanical capabilities and even exceed them in some cases [16,17,18]. The most effective strategy involves a synergistic combination of surgical intervention, post-operative care, and specifically targeted rehabilitation exercises.

Therefore, this comprehensive literature review aims to synthesize current evidence-based data to identify optimal strategies that enhance recovery, minimize complications, and improve overall functional performance in patients who experienced DBTR. Understanding the nuances of postoperative rehabilitation can provide valuable insights for both clinicians and patients, ultimately contributing to more effective management of distal biceps tendon injuries.

## 2. Methods

### Literature Identification and Selection Process

This narrative review was conducted in accordance with established methodological principles for narrative reviews, aiming to provide a comprehensive and critical synthesis of contemporary surgical techniques, fixation methods, rehabilitation protocols, and clinical/functional outcomes following DBTR. The literature search was performed utilizing the PubMed/MEDLINE databases, concentrating on studies published in English up to 19 March 2025, using the relevant keywords including “distal biceps tendon rupture”, “surgical repair”, “fixation methods”, “muscle function restoration”, “supination and flexion strength”, and “rehabilitation protocols”. In alignment with the recognized methodological guidance for narrative reviews, the search did not adhere to the formal reporting requirements of systematic reviews, including fully reproducible search strategies, predefined inclusion or exclusion criteria, or obligatory flow diagrams as required by PRISMA recommendations for systematic reviews. Instead, priority was given to randomized controlled trials, meta-analyses, and high-quality observational studies considered most representative of contemporary clinical practice and emerging evidence. Article selection was guided by relevance to the narrative scope and conceptual contribution rather than by formal eligibility criteria. No formal quality scoring, standardized data extraction forms, or quantitative synthesis were conducted, consistent with the narrative review design.

## 3. Evidence-Based Background of DBTR

### 3.1. Etiology and Risk Factors of DBTR

The biological etiology of DBTR is complex and multifactorial, including mechanical (impingement), inflammatory, degenerative, and arterial supply factors (hypovascularity of the tendon) [19]. Some risk factors are non-traumatic and include advanced age, sex (CSA and tendon-protective total estrogen levels), overweight and obesity (body mass index [BMI] > 30 kg/m^2^), chronic low-grade tendinosis, inflammation within the radial bursa, dehydration, anabolic, corticosteroid, or alcohol abuse [20], treatment with statins, and fluoroquinolones (FQs; fluoroquinolone antibiotic use can increase the risk of tendinopathy by several times) [21,22], tobacco smoking (which increases the risk of DBTR by 7.5-fold) [9], repetitive trauma with vascular compromise, corticosteroid injections into the biceps tendon, a single forced eccentric contraction of the biceps, mechanical factors affecting the biceps tendon, and abnormal insertion of the biceps tendon [23,24]. In recent years, a series of studies have found that hereditary and wild-type transthyretin-mediated (ATTR) amyloidoses, especially transthyretin-associated amyloidosis cardiomyopathy (ATTR-CM), are associated with spontaneous DBTR [25,26,27]. In addition to confirming the frequent association between ATTR-CM and DBTR, it was also established that the onset of these comorbidities typically precedes the diagnosis by several years [28]. Hence, the detection of musculoskeletal manifestations by orthopedic surgeons may enable earlier diagnosis and administration of effective treatments before disease progression occurs [25]. It is thought that spontaneous non-traumatic DBTR and any tendon rupture are infrequently observed in clinical settings and predominantly associated with long-term use of FQs (pefloxacin, ofloxacin, norfloxacin, and ciprofloxacin) or corticosteroids [29]. However, a recent systematic review identifies older age, male sex, and participation in sports as predominant risk factors for DBTRs [11].

### 3.2. Anatomical Predispositions to DBTR

Several anatomical and patient-specific factors have been identified as contributors to DBTR. The morphology of the radial tuberosity plays a key role, particularly when it is prominent or anteriorly oriented, leading to increased mechanical stress and potential impingement during forearm rotation [30]. In such cases, the tendon may be subjected to friction and shear forces at the insertion point, especially in positions of supination and flexion. Vascular supply to the distal biceps tendon is another crucial factor. A hypovascular zone near the radial insertion has been described as a site of potential tendon degeneration, reducing the tissue’s capacity to heal or withstand repetitive loading [19]. This anatomical vulnerability may explain the spontaneous rupture observed in certain populations, particularly middle-aged men who engage in high-demand physical activity. Degenerative changes at the radial tuberosity, including enthesophyte formation or cortical irregularities, are often observed in imaging of patients with DBTR and may reflect chronic traction forces or overuse [31,32]. These alterations can further compromise tendon integrity and increase the risk of avulsion under load. Patient-related risk factors also contribute to rupture susceptibility. From a surgical standpoint, the proximity of the posterior interosseous nerve (PIN) to the radial tuberosity highlights the importance of anatomical precision, as poor awareness of these relationships may increase the risk of iatrogenic injury during repair [33]. These cumulative anatomical and systemic factors, when combined with eccentric overload mechanisms, create a high-risk environment for rupture in active individuals, particularly during resisted supination or heavy lifting tasks.

### 3.3. Pathomechanism of DBTR

The mechanism of injury is characterized by a singular, unanticipated eccentric force applied to an actively flexed elbow in a pronated position [34]. Less load to failure is noted as the elbow moves toward a flexion angle of 90° [35]. The injury is common, especially in weightlifting [36,37], as evidenced by a case of a 43-year-old male who reported acute pain and weakness after a gymnastic activity, accompanied by an audible pop in his elbow [38]. The clinical presentation consists of ecchymosis and pain, often associated with physical exertion, often with no audible clicking, presenting bicipital belly rise and potentially a reverse “Popeye” sign, followed by functionally significant strength loss for elbow flexion and forearm supination (30% and 40% strength loss, respectively), and decreased resistance to fatigue [1,2,7,39]. Surgical repair in this specific injury generally focuses on regaining function, i.e., ROM, forearm supination, and elbow flexion strength, and complication risks, resulting in a good clinical and functional outcome [40,41].

## 4. Surgical Intervention and Repair Techniques

Surgical intervention is often the preferred treatment for DBTR, especially in active individuals, to restore muscle function. Comparative studies indicate better functional results with surgical treatment, despite an overall complication rate of 4.6–25% [40,42,43]. The ideal timing for surgery is within 1 to 2 weeks post-injury to minimize scar tissue formation [44]. Several techniques have been described for the surgical treatment of DBTR, including single anterior incision, often complicated by a high incidence of radial nerve palsy, and double-incision techniques exposing the radial tuberosity and allowing a smaller anterior approach, often complicated by frequent post-operative proximal radio-ulnar synostosis [41,45,46]. Importantly, the precise choice of surgical technique can significantly influence long-term clinical and functional outcomes [47,48]. The surgical technique used can be selected based on the patient’s clinical presentation, the condition of the tendon, the patient’s bone anatomy, and the surgeon’s skills and experience. Both single- and double-incision techniques have been extensively examined [45,49]. Recent reports present results of various modifications and improvements in the previously described techniques. Each technique has advantages and disadvantages, but no difference was found in functional outcomes between single- and double-incision procedures, with complication rates being 23–26% [50]. Therefore, no specific surgical technique can be deemed the best treatment for all patients, as in terms of preventing post-operative complications, they have largely equilibrated and are similar between groups using modern surgical techniques [50,51]. Moreover, the ideal fixation method (transosseous suture, suture anchors, interference screws, or cortical buttons) has also been debated [52]. The patient-specific surgical treatment protocol is based on the concept that the ideal treatment should provide coverage of the ruptured tendon stump with surrounding soft tissue, be technically simple, be minimally invasive, protect the blood vessels at the tendon attachment site, and decrease the chance of nerve injury. The ideal treatment allows a short rehabilitation period, defined as early controlled mobilization with active or assisted motion initiated within the first 1–2 postoperative weeks [53].

For the anatomic reconstruction of DBTR, a universally accepted surgical methodology has yet to be delineated. The presently available surgical techniques aim to achieve reinsertion at the osseous footprint of the radial tuberosity, thereby reinstating tendon-surface contact [34]. Various distal biceps tendon repair methodologies, including intraosseous tendon placement and onlay techniques, have demonstrated remarkable restoration of biomechanical properties in comparison to the native tendon [6,54]. Anatomic reconstruction is highly recommended, particularly for athletes or individuals who rely heavily on supination strength and endurance. This technique ensures significantly better supination and flexion strength [55,56]. Given the patient-specific factors, anatomic repair is particularly advisable for elite athletes, as it restores greater supination peak torque and fatigue strength, which are critical for high-performance activities [55,56]. The cortical button technique is widely used for its strength and reliability. Subtle variations in drill hole positioning can achieve either anatomic or non-anatomic repairs, with anatomic repairs yielding superior supination and flexion fatigue strength [55]. In cases of chronic ruptures or significant tendon loss, tendon grafts (e.g., Achilles allografts) may be necessary [57]. However, these cases may result in weaker supination strength compared to primary repairs [58,59].

### 4.1. Single-Incision Repair

The single-incision (SI) technique, often utilizing suture anchors or cortical buttons, has shown promising results in restoring strength and endurance. Studies indicate that SI repair achieves excellent functional outcomes, with patients regaining near-normal flexion and supination strength [60]. For instance, the SI approach has been found to yield better supination recovery than the double-incision method, likely due to reduced disruption of surrounding tissues and muscles [45,61].

The major disadvantage of the SI technique is the difficulty in visualization, especially when dealing with the lacertus fibrosus, which is often present in these cases [49]. Furthermore, the subcutaneous space is highly contaminated, making the possibility of superficial infection more likely, which would be difficult to manage owing to the prosthesis passing through the area. Finally, there is the problem of identifying and localizing the disrupted biceps and the muscles of the ventral compartment. These factors make SI repairs not up to modern surgical techniques. Superficial exposure of the joint only at a point defined by the conjoint tendinous structure adds an unnatural traction that would not result from conventional surgical incisions.

We have been unable to find either clinical or cadaveric studies that disprove or perhaps invalidate SI approaches. The single-incision technique would be feasible if a laparoscope or endoscope could visualize the area. Minimally invasive and endoscopic-assisted techniques have received increasing attention in recent years, reflecting growing clinical and academic interest [62,63,64]. The potential benefits of minimally invasive approaches, such as low complication rates, reduced soft-tissue dissection, and faster recovery, warrant further investigation.

### 4.2. Double-Incision Repair

Boyd and Anderson [65] described this technique, which was later modified by Morrey et al. [66]. The procedure, which involves bone-tunnel fixation, demonstrates strong outcomes in restoring strength, endurance, and motion, with a low rate of postoperative complications (e.g., neurological).

In double-incision (DI) repair, portions of the distal tendon are passed through two separate incisions in the distal attachment and sutured in one or two layers. The operation thereby becomes longer and more invasive, which leads to a higher risk of damaging the highly perfused structures, resulting in worse post-operative pain and a need for longer immobilization and post-operative rehabilitation in athletes [67,68]. The displacements of the individual threads can lead to a partial tear of the correctional suture. If they are noticed in the late stage postoperatively, the “second stage” operation is necessary again. During the reconstruction of tendon lesions, two parts of the tendon are drilled individually and connected to the attachment by being tied through the bone drill holes in a double incision above the distal attachment and under the attachment.

### 4.3. Comparative Analysis: Single-Incision Versus Double-Incision Technique in DBTR

While numerous surgical techniques have been described with overall good results [49,69,70], there are still debates about the choice between SI and DI approaches [71,72,73], and the type of tendon fixations [6,61,74,75,76].

A randomized clinical trial highlighted a 10% advantage in final flexion strength for the DI method compared to SI, though this difference was not clinically significant in most cases [45]. Another study found that the DI approach provided excellent return of elbow functionality, with low rates of neurological complications [77]. Karunakar et al. [78] reported 48% elbow weakness in supination, compared to 14% for flexion, and loss of endurance in 38 elbows following the DI technique. These values were derived from side-to-side comparisons within the same patients. Moreover, Jobin et al. [3] confirmed that a DI approach can more closely recreate the anatomical footprint than the SI approach. Dunphy et al. [42] conducted a comparative analysis of DI and SI surgical repairs. They observed a statistically significant elevation in the incidence of posterior interosseous nerve (PIN) palsy (3.4% vs. 0.8%, *p* = 0.010), heterotopic bone formation (7.6% vs. 2.7%, *p* = 0.004), and the necessity for reoperation (8.3% vs. 2.3%, *p* < 0.001). As mentioned previously, the surgical intervention for DBTRs is characterized by a low incidence of severe complications, irrespective of the surgical approach or technique employed. Nonetheless, the DI technique is associated with a greater incidence of PIN palsy, heterotopic bone formation, superficial wound infection, and the requirement for reoperation. In general, comparative studies suggest that while both SI and DI techniques yield favorable outcomes, the choice of technique should be tailored to patient-specific factors, such as the risk of complications and the surgeon’s expertise [49,69,70]. Complications, such as reruptures and nerve-related injuries (neurapraxia, neuritis, paresthesia, and dysesthesia), are relatively rare but more common in certain surgical techniques. Complication rates have been reported to be 5–63% [79,80]. Possible complications should be carefully managed to ensure optimal outcomes, but most patients experience transient and manageable side effects [49,69,81]. For example, re-rupture occurs in less than 5% of cases (even 1.5% after primary repair) [43,82], although one database study reported a 5.4% re-rupture rate without distinguishing between acute and chronic cases [83]. When it does occur, it is often due to inadequate fixation or premature return to heavy activity [84]. In line with this, others also have pointed out a higher risk of re-rupture, especially if the repair/reconstruction is non-anatomic or if the patient returns to high-demand activities too early [55,58]. Some patients may experience injuries to the PIN and transient paresthesia of the cutaneous nerve, which typically resolves on its own [58]. However, sensory neurapraxia after distal biceps tendon repair is not associated with patient-reported outcomes or satisfaction [85].

### 4.4. Post-Operative Complications

In conjunction with numerous high-quality randomized clinical trials (RCTs) or retrospective cohort studies, several recent systematic reviews and meta-analyses have been published concerning the complications of surgical techniques in patients post-DBTR. According to Kodde et al. [86], a systematic review indicated reduced complications associated with the DI approach employing bone tunnel fixation. This approach exhibited a markedly lower incidence of complications than the SI anterior approach, and the bone tunnel fixation had significantly fewer complications than the 3 alternative fixation techniques (suture anchors, interference screws, and cortical buttons). However, as the DI approach was used with bone tunnel fixation in 84% of cases, there was a strong interrelationship between these variables. A meta-analysis of Castioni et al. [71] assessed the outcomes of SI versus DI, including final ROM, the Disabilities of the Arm, Shoulder, and Hand (DASH) score, and neurological and non-neurological complications. The findings indicated that SI resulted in superior final ROM for flexion and pronation, reduced rates of heterotopic ossification and reoperations, and increased risk of lateral antebrachial cutaneous nerve (LACN) paresthesia due to a more extensive dissection and longer duration of deep anterior retractor placement. Importantly, all differences were statistically significant.

In the most extensive analysis concerning complications following DBTR, the overall incidence of complications was established at 25%, with the major complication rate documented at 4.6% [40]. Specifically, among the 774 distal biceps repairs, major complications comprised a 1.6% (*n* = 51) occurrence of PIN injury; a 0.3% (*n* = 10) incidence of median nerve injury; a 1.4% (*n* = 43) rate of rerupture; and a 0.1% (*n* = 4) incidence of synostosis, which was exclusively observed with the DI technique. Notably, the choice of fixation technique did not significantly impact the rates of rerupture and PIN injury. Others have similarly delineated a modified DI technique, incorporating a muscle-splitting technique via the common extensor of the digits. More contemporaneously, with the emergence of enhanced methodologies and implants, including suture anchors, intraosseous screws, and suspensory cortical buttons, SI techniques have experienced a resurgence in favor [87,88].

### 4.5. Non-Operative Treatment

The current literature supporting non-operative management comprises mostly retrospective Level III–IV cohort studies, case series, systematic reviews, and meta-analyses of heterogeneous, often low-level studies; definitive randomized data are lacking [48,89,90]. Interpretations should therefore be cautious and individualized. Conservative management may be considered for patients with lower physical demands, though surgical repair is generally recommended for optimal outcomes [55]. Some studies on operative success have reported that functional outcome scores and disability can remain the same for both operative and non-operative groups [8,91]. There is an increasing volume of research suggesting that non-operative treatment yields acceptable outcomes for those affected, thereby offering an alternative to current practice [14,92,93,94]. For example, a retrospective cohort study found no difference in functional outcomes and strength scores between operative and non-operative patients [92]. However, non-operative care is designated for patients averse to surgery, tolerant of functional and strength limitations, medically compromised, with partial tears (affecting <50% of the tendon), or with chronic rupture [95]. Although still, non-operative treatment is rarely recommended, if selected, rehabilitation should focus on strength enhancement rather than endurance [96]. Because most comparisons are non-randomized and subject to selection bias (operative patients are often younger or higher-demand), reported differences in strength or complications may partly reflect baseline differences rather than treatment effects [89].

## 5. Rehabilitation Protocols Following DBTR

Time-based (dependent on tissue healing) and criterion-based rehabilitation programs/protocols have evolved with advancements in surgical techniques, emphasizing the importance of communication between the physician and rehabilitation specialist to tailor the recovery process to individual patient needs [96]. It is challenging to ascertain any unequivocal correlation between the surgical technique and the subsequent rehabilitation protocol administered to patients post-surgical anatomical reattachment of the distal biceps tendon [97]. A notable deficiency exists in the literature concerning physiotherapy or rehabilitation program types and the necessity of therapy post-DBTR. Effective rehabilitation protocols often incorporate a combination of physical therapy, progressive loading exercises, and patient education to ensure a comprehensive approach to recovery. These elements work synergistically to facilitate healing, restore mobility, and rebuild strength in the affected arm while addressing individual patient needs and goals throughout rehabilitation [98]. Tailoring these rehabilitation protocols to each patient’s unique circumstances can lead to better outcomes, as it allows for adjustments based on factors such as age, activity level, comorbidities, and the severity of the injury (e.g., partial versus complete DBTR) [6,99].

### 5.1. Early Mobilization Versus Immobilization

A sequential, progressive, and multi-phased rehabilitation approach is widely recommended, commencing with post-operative joint immobilization for 3–6 weeks followed by gradual mobilization [17]. A universal post-operative protocol includes gentle exercises to maintain joint mobility without overloading the repaired tendon [55]. Gradually increasing resistance exercises should be introduced to improve flexion and supination strength. In general, gradual strengthening of the upper extremity and aerobic conditioning may begin at 6 weeks post-surgery, with return to normal levels of activities, including sports, at 12–20 weeks [7,100]. Gentle progression of exercises is aimed at a smooth formation of the absent tendon in the stages of uncontrolled passive and active movements of the retrained limb of the patient in the immediate postoperative period. The cytoprotectant therapy with preparations (pharmacological agents modulating early inflammatory responses) promotes the elimination of the initial destructive phase of healing and excessive formation of adhesions. Such therapy and its further continuation negatively influence the trophism of the regenerative scar, and it should be prescribed only in dystrophic cases with the presence of their clinical indices [101,102]. In addition, this approach minimizes the risk of re-rupture while promoting optimal recovery of muscle function (strength, ROM, and endurance) [17,55,58]. However, early mobilization protocols, which involve early active range of motion (AROM) and strengthening exercises within the first 2–3 postoperative weeks, have enhanced functional outcomes, reduced the risk of stiffness, and promoted tendon healing [103,104]. From a rehabilitation perspective, early mobilization does not compromise tendon integrity and accelerates return to light daily activities and non-manual work by approximately 2–4 [17,104]. A randomized controlled trial comparing early mobilization with immobilization found no significant differences in strength or ROM, yet patients in the early mobilization group had superior QuickDASH (the shortened version of the DASH) scores, indicating improved functional outcomes [104]. This suggests that early mobilization can be integrated into rehabilitation protocols without compromising strength retention. On the other hand, a recent systematic review and meta-analysis found no clinically significant differences in patient-reported outcomes and ROM between early and delayed mobilization after primary distal biceps tendon repair [105]. It has been proposed that unrestricted or early ROM may commence earlier since repair strength is greater than the force of an unweighted forearm in a splint or brace [56,106]. Smith and Amirfeyz [107] did not encounter any failure of the repair using an unrestrictive early mobilization rehabilitation program. The cohort achieved an average of 99% ROM of the uninjured arm, with no pronosupination deficits at a mean follow-up of 16.6 months.

Therefore, the implementation of supervised functional training is advocated within the rehabilitative framework for individuals who have experienced DBTR. A comparative study of supervised versus unsupervised therapy post-repair facilitated patients’ immediate use of the operated limb for daily activities [108]. Incorporating task-specific exercises that mimic daily activities or occupational demands ensures that the rehabilitation program is patient-centered and relevant to the individual’s lifestyle [109]. Manual isometric resistance exercises of the two forearm muscles are introduced according to the patient’s specific pain. Chronic pain may persist at the site of injury, and it may be exacerbated by physical effort; a weakness in grip strength could also remain. A disseminated post-operative protocol has restricted load-bearing to 5–10 pounds during the initial weeks and has constrained M. biceps brachii isotonic exercises until the 12th week. However, such protocols exhibit variability among different surgical practitioners. The site of reattachment is particularly susceptible to failure within the first 1–2 weeks post-surgery [7].

Resistance exercises involving muscle groups, active joint participation, and eccentric and concentric exercise rehabilitation actions are included in the compressive force of the short head of the M. biceps brachii. Functional exercise with only the long portion of the M. biceps brachii, related to the required work and symptomatology, is indicated. Resisted exercises are proposed after two manual activities without symptoms, since excessive unloading or avoidance of muscle activation may lead to muscle atrophy [110]. Exercise overload depends on associated injuries, age, muscle strength, and specific tasks, with an adaptation period lasting 4–5 months.

Strenuous, gradual resistance exercises during rhythmic or cyclical exercises can be introduced once normalizing agility or full ROM has been achieved [111]. However, these exercises should not generate pain or tension in the harvest area or present a risk of excessive use. The first movement to be incorporated in this stage will be the exercise of sustained vibration adopted by the antigravity and eccentric muscle strengthening cycle. The progressive resistance must be performed without pain and stopped if muscle fatigue is detected. The abundance of elongated muscle tissue and the elimination of scar tissue occur throughout the first post-injury year. Care is recommended, especially in muscle overload situations, such as weightlifting, when muscle regeneration and quality are not homogeneous.

Standardized home-based rehabilitation programs, including gradual strengthening and ROM exercises, have effectively restored normal elbow function. These protocols are particularly beneficial for patients who may not have access to formal physical therapy [112]. Moreover, endurance of the remaining intact musculotendinous units does not seem to increase or decrease over time [56].

### 5.2. Post-DBTR Flexion and Supination Strength Retention

Strength retention is a critical outcome measure following DBTR. Complete DBTR results in substantial reductions in elbow flexion and forearm supination strength. Strength assessments consistently reveal that a majority of individuals recover a significant proportion of their pre-injury muscular strength after surgical intervention, particularly in elbow flexion and supination [113], although minor deficits may persist. Empirical data concerning strength recovery after anatomic repair show that flexion strength reaches up to 90% and supination strength up to 78% of the non-operated side [55,59].

Several patient-specific factors influence strength retention, i.e., age, activity level, dominance, and compliance. Younger, active patients tend to retain better strength after DBTR. A study focusing on this population reported that 88% of patients regained more than 90% of their original strength in supination and flexion [114]. Non-compliance can lead to suboptimal outcomes, while adherence to rehabilitation protocols can maximize strength recovery [104].

Restoration of flexion and supination strength in adult cohorts following distal biceps tendon reinsertion varies among studies. Most previous studies showed that the loss of flexion strength is about 10–20%, but tends to improve over time [2,115,116]. Clinical trials report that elbow flexion strength is restored to 94–102% of the uninjured side. For instance, one study found that at an average follow-up of 3.7 years, flexion strength was 96% of the contralateral side [117]. Another study using the ToggleLoc technique reported flexion strength recovery of 101% at 5-year follow-up [118]. Supination strength is also largely restored, though deficits may be slightly more pronounced. McCarty et al.’s [119] analysis revealed that non-operatively treated DBTR results in approximately 40% supination strength and 47% endurance loss. Previous reports also indicated a 20–30% loss of supination strength without repair [120]. Table 1 presents clinical and surgical factors associated with recovery of peak strength.

Cortical button fixation is a popular method for DBTR, offering strong biomechanical stability and anatomical reinsertion. Studies have shown excellent functional outcomes, with patients achieving near-normal supination strength and minimal complications such as heterotopic ossification [64,128,129]. A study using cortical button fixation reported supination strength at 91% of the uninjured side [117], while another study using the Endobutton technique achieved 99% recovery [130]. Similar clinical effects were observed by Greenberg et al. [131], who found that patients treated with Endobutton experienced a 97% recovery of flexion and 82% of supination strength compared to the opposite arm at 20 months. It was confirmed that the Endobutton fixation has the highest load resistance and stiffness among all fixation methods [131], although this superiority does not translate into better clinical results [132]. The peak failure load range of Endobutton is reported to be 270–440 [133,134]. We believe that case–control studies comparing the function of surgically treated DBTR with the healthy contralateral limb are more effective in assessing patient satisfaction.

Isokinetic and isometric dynamometers are devices that provide objective measurements of strength recovery, allowing for precise tracking of progress [55,58]. Isokinetic testing revealed that endurance may be slightly reduced while strength is largely restored, particularly in supination. For instance, isokinetic testing revealed a 40–50% reduction in supination strength and a decrease of over 60% in endurance at prolonged flexion [115]. Another study found that supination endurance was 20% less in the dominant extremity of athletes [135]. A recent systematic review and meta-analysis highlights that surgical repair leads to significant improvements in flexion and supination strength, with isokinetic flexion strength showing a reduction of −4.56 Nm and supination strength a reduction of −1.18 Nm compared to the healthy arm. Overall, while functional outcomes are slightly inferior to the contralateral arm, surgery is considered to yield good functional results [15].

In addition, recent advances in technology (e.g., exoskeletons with assist-as-needed algorithms) have provided innovative tools to monitor the rehabilitation process and enhance outcomes [126]. Bio-inspired exoskeletons equipped with force-sensitive resistors and machine-learning algorithms can provide personalized assistance during exercises. These devices offer real-time feedback on progress, which can motivate patients to adhere to their rehabilitation programs [109]. Electromyography (EMG) feedback is another essential tool in rehabilitation methodology. EMG signals from the M. biceps brachii can be used to monitor muscle activity and provide patients with real-time feedback, helping them understand their progress and engage more effectively in therapy sessions [109].

### 5.3. Return to Work and Play

The average time to return to sports or full activity after surgery is approximately 5.4 months, although this can vary depending on the patient’s condition and the specific rehabilitation protocol followed [136]. Patient-reported functional outcomes, measured using scores such as the Mayo Elbow Performance Score (MEPS) and DASH questionnaire, are consistently high following DBTR. One study reported a mean DASH score of 6.3, comparable to the general population [133]. Patients often achieve near-normal supination and flexion strength, with minimal residual deficits [70,128,129].

Most patients, including athletes, can resume their previous activities without significant limitations. Studies report that even 93–100% of patients return to their previous level of physical activity, including competitive sports. For example, one study found that all athletes returned to full, unlimited activity, with excellent subjective results [133]. Other studies showed that 84–94% of National Football League (NFL) players returned to play in 7–15 months after DBTR, with no difference in performance compared to pre-injury levels [56,137]. Strength training usually commences about 2–3 months post-operative [135]. Patients typically return to their baseline functional capacity levels within 6 months with excellent clinical outcomes [138,139,140,141]. Even 97.5% of the athletes can successfully return to their former sport activity in a period of <9 months [74]. A prior study investigated post-surgical physical activity, revealing that 80% of patients returned to their pre-injury activity levels. The average time to resume training was 12.05 weeks, while full activity was achieved at 36 weeks [141]. Concerning the specifics of physical training, return to activities such as heavy lifting or high-impact sports is allowed at 3–6 months after surgery [6]. Recently, Pitsilos et al. [68] found a relation between the surgical technique and time of return to sport. An earlier return to sport was associated with nondominant-side (*p* = 0.007) and acute (*p* < 0.001) injuries, participation in weightlifting (*p* = 0.001), a DI approach (*p* = 0.005), cortical button fixation (*p* < 0.001), and the absence of supination-pronation restriction (*p* = 0.032).

### 5.4. Acute and Chronic DBTR

The timing of intervention is another area that warrants further investigation. Distal biceps tendon ruptures have been classified as chronic between 2 and 6 weeks after injury. However, Bajwa et al. [142] defined chronic DBTRs as ruptures persisting beyond 21 days of injury. Chronic DBTR often presents unique challenges due to tendon retraction and fibrosis, but surgical reconstruction is still a viable option. Research indicates that acute and chronic DBTR result in comparable patient satisfaction, functional outcome scores, and adequate ROM [130]. While acute repairs typically restore functionality, chronic cases may require reconstructive techniques due to tendon retraction and scar formation [119]. However, chronic repairs may have a slightly higher risk of transient lateral antebrachial cutaneous nerve (LABCN) injury palsy than acute cases [70,81]. The repair timeline for the distal biceps tendon to prevent complications ranges from 4 to 12 weeks after rupture [143,144]. Early intervention is generally recommended to minimize complications and optimize recovery. Primary repair might be recommended in young active patients because of the risk of permanent deformity and potential interference with forearm rotation that might occur with a DBTR [145]. However, even in chronic ruptures, surgical reconstruction can restore full function, as demonstrated by a case where a patient regained full function 13 years post-injury [59]. Late anatomical repair can yield very good flexion and moderate supination strength, even when the primary diagnosis is delayed [59]. The recent case–control study found no significant difference in strength recovery (89.19% in flexion and 77.48% in supination) between groups, regardless of time to surgery [70]. There was also no significant difference in the MEPS, Q-DASH, and the Patient-Rated Elbow Evaluation (PREE) functional scores between the two cohorts (*p* = 0.354, *p* = 0.412, and *p* = 0.958, respectively). Long-term follow-up studies have demonstrated the durability of DBTR, with patients maintaining functional gains and experiencing minimal recurrence of symptoms. The selection of surgical technique and corresponding rehabilitation protocol considerably influences the long-term prognostic results [47,48]. Moreover, Achilles tendon allografts have been successfully used in chronic cases, with outcomes showing full functional recovery and a DASH score of zero at 12-month follow-up [65]. According to Frank et al. [146], delayed reconstruction of irreparable DBTRs with semitendinosus autograft produces similar strength, ROM, and complication rates but slightly worse functional outcome scores compared with delayed primary repair. Synovec et al. [147] compared outcomes of autograft and allograft reconstructions, finding that allografts had lower complication rates, particularly donor site morbidity associated with autografts. Additionally, the development of novel graft materials, such as dermal matrices, could offer promising alternatives for chronic ruptures [148]. It is also important to note that a slow and incomplete functional recovery of the forearm characterizes this type of lesion. Non-union cases are frequent, and reattachment of the DBTR or supination/flexion rehabilitation to the side may be necessary. Unrepaired M. biceps brachii tendons that are allowed to remain unattached have little functional consequence other than a cosmetic defect. It can also cause a decrease in a specific performance skill.

### 5.5. Future Directions in Research

There is a paucity of biomechanical studies in the literature that clearly define the use and need for the distal biceps tendon for functional activities and/or specific functional activities. The distal biceps utilizes an alternative torque on supination of the forearm, supinating the elbow at a fixed angle with the shoulder abducted to 90 degrees. It is not used in this motion unless fatigue occurs in the other elbow supinator muscles. It is also interesting to identify at what age and why DBTRs occur in patients and to compare these two groups. It would also be of value to the patient and surgeon to have better and more reliable data that allow the surgeon to identify “high risk.” Individual patients or groups of patients who could benefit from reattachment or reconstruction, or, conversely, where they would not need surgery. Given the uncompromised nature of the contralateral biceps in the majority of the population, it is interesting that no study to date has looked at how the overall function and capacity of an individual’s M. biceps brachii may influence the likelihood of rupture.

## 6. Conclusions

Anatomic repair is favored over non-operative methods due to enhanced results, especially for athletes and individuals requiring significant supination strength. Chronic cases should not be dismissed, as surgical reconstruction can still yield excellent results. The optimal rehabilitation protocol for restoring muscle function in patients with a DBTR involves a combination of surgical intervention, early mobilization, progressive resistance exercises, and the use of advanced technologies for personalized feedback. Tailoring the rehabilitation program to the patient’s specific needs ensures the best possible functional recovery and return to daily activities or competitive sports. Long-term outcomes of patients post-DBTR often show variability in strength retention and range of motion, indicating the fragility of statistical findings in these surgical interventions.

## Figures and Tables

**Table 1:** Reported clinical and surgical factors associated with recovery of peak strength.

Citation	Insights	Population Sample	Dependent Variables	Results
Barret et al. [103]	Key factors influencing the restoration of peak strength following distal biceps tendon reinsertions include the surgical technique (double incision), immediate postoperative mobilization, and the patient’s activity level, which collectively contribute to reduced complications and improved functional outcomes.	▪ Sample size: 74 men with acute distal biceps tendon tears. ▪ Sampling method: Retrospective single-center study.	▪ Pain evaluated using the VAS scale. ▪ Range of motion and strength measurements.	▪ Mean pain score on VAS was 0.22. ▪ 94% strength in flexion, 90.5% in supination.
Morrey et al. [121]	The study indicates that primary repairs performed in high flexion (60–90 degrees) can yield excellent outcomes without significant complications. Additionally, the ability of the biceps muscle to lengthen over time contributes to strength restoration, minimizing the risk of rerupture or flexion contracture.	▪ Sample size: 188 distal biceps tendon repairs ▪ The sampling method involved a retrospective case–control study design	▪ Allograft augmentation in distal biceps tendon repairs ▪ Functional outcomes were evaluated using the Mayo Elbow Performance Score (MEPS)	▪ All patients had a Mayo Elbow Performance Score of 100. ▪ Complications included 3 neurapraxias and 1 rerupture.
Faict et al. [122]	Key factors influencing the restoration of peak strength following distal biceps tendon reinsertion include the surgical technique used, the timing of the intervention after conservative treatment, and the patient’s adherence to rehabilitation protocols post-surgery.	▪ Sample size: 20 patients with distal biceps tendinopathy. ▪ Sampling method: Retrospective cohort study design.	▪ Quick-Dash score ▪ Liverpool Elbow Score ▪ Mayo Elbow Performance Index ▪ Broberg and Morrey Score ▪ Short HSS Scoring System ▪ Isokinetic strength testing	▪ Good functional outcomes after distal biceps tendon surgery. ▪ No significant differences in strength between operated and non-operated sides.
Gasparella et al. [123]	Key factors influencing the restoration of peak strength following distal biceps tendon reinsertions include the timing of surgery (delay increases complications), patient age, history of smoking, rheumatologic conditions, and the use of anabolic steroids, which affect functional recovery.	▪ Sample size: 14 male patients. ▪ Sampling method: Consecutive treatment by one author.	▪ DASH score questionnaire ▪ MEPS questionnaire ▪ Isokinetic biomechanical tests	▪ Mean DASH score: 4.7 points, excellent MEPS: 96.8 points. ▪ Average flexion strength increase: 10.2% compared to unoperated arm.
Murena et al. [124]	Key factors influencing the restoration of peak strength following distal biceps tendon reinsertions include the surgical approach used, with a mini-invasive approach showing a positive trend in strength recovery compared to the standard extensile approach. Additionally, the time to return to sports and work, which averaged three months postoperatively, plays a crucial role. The study reported a 93% full return to activities, indicating that effective rehabilitation and surgical technique significantly impact strength restoration outcomes.	▪ Sample size: 28 cases of biceps tendon rupture, which were evaluated at a mean follow-up of 45 months. ▪ The sampling method involved two groups: 19 cases underwent a standard anterior approach (Group A), while 9 cases were treated using a less invasive anterior approach (Group B).	▪ Elbow ROM was assessed to evaluate the functional recovery of patients after distal biceps tendon reconstruction, with a mean recovery reported as almost complete. ▪ Subjective strength recovery was measured to determine the ability of patients to return to sports and work activities.	▪ The mean ROM recovery was almost complete, with 93% of patients achieving full return to sports and work within an average of 3 months postoperatively. ▪ The complication rate was significantly lower in the mini-invasive approach group (Group B) at 0%, compared to 26% in the standard extensile approach group (Group A).
Darlis [125]	The restoration of peak strength following distal biceps tendon reinsertions is influenced by several key factors: the anatomical reinsertion of the tendon to the bicipital tuberosity, the technique used (such as the proposed posterior to anterior method), the quality of fixation (utilizing two sutures and an endo-button), and the timing of rehabilitation (active motion starting at three weeks postoperatively). Additionally, minimizing complications like heterotopic ossification and ensuring adequate tendon-to-bone contact are crucial for optimal recovery.	▪ Sample size: 38 male individuals who had acute distal biceps ruptures, specifically those who were treated within one month of their injury. ▪ The sampling method involved applying the new anatomical reinsertion technique to these individuals, which can also be modified for chronic ruptures using allograft or autograft.	▪ The study evaluated the healing outcomes of the new biceps tendon reinsertion technique, precisely measuring ROM and supination strength in patients postoperatively. ▪ The incidence of complications such as re-rupture and heterotopic ossification was also monitored to assess the technique’s effectiveness and safety.	▪ The new technique resulted in one early re-rupture due to suture failure and one case of heterotopic ossification that required re-operation. ▪ The majority of patients healed uneventfully, achieving full ROM and returning to their previous occupations and athletic activities with M5 supination strength at manual testing, indicating successful restoration of function and strength.
Alech-Tournier et al. [126]	Key factors influencing the restoration of peak strength following distal biceps tendon reinsertions include the timing of surgery, the technique used (such as ToggleLoc™ with ZipLoop™), and the degree of tension applied during the reattachment procedure.	▪ Sample size: 38 patients ▪ Retrospective study design used for sampling	▪ Mean strength deficit in supination ▪ Instances of heterotopic ossification and nerve paresthesia	▪ Mean strength deficit in supination: 23.9% compared to contralateral side. ▪ Four cases of heterotopic ossification observed.
Khwaja et al. [127]	The paper does not specifically address key factors influencing the restoration of peak strength following distal biceps tendon reinsertions. It focuses on patient-reported outcomes, function, complication rates, and radiographic findings related to the dual incision cortical button technique.	▪ Sample size: 22 patients participated in the study. ▪ Sampling method: Single surgeon standardized technique for all patients.	▪ Patient-reported outcomes (DASH, Oxford, Mayo scores) ▪ Range of movement and strength measurements	▪ Good clinical outcomes with no major complications. ▪ Heterotopic ossification affected supination strength in some patients.

## Data Availability

No new data were created or analyzed in this study. Data sharing is not applicable to this article.

## Abbreviations

The following abbreviations are used in this manuscript:

DBTR	Distal biceps tendon rupture
CSA	Cross-sectional area
ROM	Range of motion
BMI	Body mass index
FQs	Fluoroquinolones
ATTR	Wild-type transthyretin-mediated
ATTR-CM	Transthyretin-associated amyloidosis cardiomyopathy
PIN	Posterior interosseous nerve
SI	Single-incision
DI	Double-incision
RCTs	Randomized clinical trials
DASH	Disabilities of the Arm, Shoulder, and Hand
LACN	Lateral antebrachial cutaneous nerve
AROM	Active range of motion
EMG	Electromyography
MEPS	Mayo Elbow Performance Score
NFL	National Football League
PREE	Patient-Related Elbow Evaluation
